# A Label-Free Liquid Crystal Biosensor Based on Specific DNA Aptamer Probes for Sensitive Detection of Amoxicillin Antibiotic

**DOI:** 10.3390/mi12040370

**Published:** 2021-03-30

**Authors:** Duy Khiem Nguyen, Chang-Hyun Jang

**Affiliations:** Department of Chemistry, Gachon University, Seongnam-daero 1342, Sujeong-gu, Seongnam-si, Gyeonggi-do 13120, Korea; khiem80@gachon.ac.kr

**Keywords:** liquid crystals, liquid crystal biosensors, amoxicillin, DNA aptamer, polarized light microscopy

## Abstract

We developed a liquid crystal (LC) aptamer biosensor for the sensitive detection of amoxicillin (AMX). The AMX aptamer was immobilized onto the surface of a glass slide modified with a mixed self-assembled layer of dimethyloctadecyl [3-(trimethoxysilyl) propyl] ammonium chloride (DMOAP) and (3-aminopropyl) triethoxysilane (APTES). The long alkyl chains of DMOAP maintained the LC molecules in a homeotropic orientation and induced a dark optical appearance under a polarized light microscope (POM). In the presence of AMX, the specific binding of the aptamer and AMX molecules induced a conformational change in the aptamers, leading to the disruption of the homeotropic orientation of LCs, resulting in a bright optical appearance. The developed aptasensor showed high specificity and a low detection limit of 3.5 nM. Moreover, the potential application of the developed aptasensor for the detection of AMX in environmental samples was also demonstrated. Therefore, the proposed aptasensor is a promising platform for simple, rapid, and label-free monitoring of AMX in an actual water environment with high selectivity and sensitivity.

## 1. Introduction

Amoxicillin (AMX) is a semi-synthetic, penicillin-type antibiotic commonly used for the treatment of infectious diseases, such as otitis media, pneumonia, and urinary tract infection, due to its strong bactericidal capabilities and low cost [1,2,3,4,5,6]. However, when used excessively, AMX can cause side effects or adverse reactions, including dermatitis, acute interstitial nephritis, panic disorder, nausea, and diarrhea [1,7]. Moreover, the abuse or improper use of antibiotics for extended periods can promote bacterial resistance, reducing their antimicrobial effectiveness [6,8]. This can also be applied to their overuse in food-producing animals, which can lead to the presence of undesirable residues in food and antibiotic accumulation in water sources, highlighting their potential as an environmental pollution hazard [9,10,11]. Thus, the effective removal of antibiotics from aquatic environments is receiving increasing attention from governments, as well as regulatory authorities, with the detection of AMX in water representing a crucial step for its removal [10,12].

Several methods have been presented to detect AMX, such as high-performance liquid chromatography (HPLC) [13], surface plasmon resonance [14,15], colorimetry [16], fluorescence [10], and electrochemistry [12,17]. However, the need for cumbersome and expensive instruments, highly skilled personnel, complex sample preparation, and time-consuming procedures limits their application. Therefore, the development of a simple, effective, and sensitive analytical method for monitoring AMX is highly desirable. 

Liquid crystals (LCs) are attractive materials that possess various unique properties, such as optical anisotropy, birefringence, orientational behavior, and long-range orientation [18,19,20]. The orientation of LC molecules is highly sensitive to external stimuli at the molecular level [21]. The binding of molecules to the surface may induce orientational changes in the LC molecules that can be further transduced into optical signals visible using a polarized light microscope (POM) [22,23]. Therefore, LC-based biosensors have been widely applied in chemical and biological analyses and environmental monitoring [21,24,25,26]. LC-based biosensors are label-free, highly sensitive platforms and possess a simple structure, easy operation, and low cost [27,28]. Moreover, LC-based biosensors are considered promising devices that are portable through smartphone-integrated microscopes [29,30]. These developments have made way for new perspectives and opportunities for the practical application of LC portable sensing devices in the commercial market [30].

In recent years, aptamers in LC-based biosensors have been rapidly developed because of their excellent ability to recognize and bind to the target molecules, enhancing the selectivity of biosensors [1,26,27]. Aptamers, single-stranded DNA or RNA sequences, are ideal molecule recognition probes for the design and fabrication of biosensors [18,23]. They possess distinctive properties, such as ease of synthesis, high affinity, high specificity and stability, cost-effectiveness, and wide applicability [25,29,31]. Thus, LC-based aptasensors have been applied for the determination of various analytes, including sulfadimethoxine [18], pesticides [24], polychlorinated biphenyls [29], ractopamine [32], hormones [33], proteins [22,28,34], antibiotics [23,35], and heavy metal ions [25,26,36,37].

Here, we developed a simple and sensitive LC-based aptasensor for the detection of AMX in aqueous solutions. In the developed biosensor, the surface of the glass substrates was modified with a DMOAP monolayer to align LCs in homeotropic orientation, providing a uniformly dark background. AMX aptamers immobilized on the glass substrates acted as target recognition probes to capture AMX molecules. The specific binding of aptamers and AMX molecules results in conformational changes in aptamers, inducing an orientational change in LCs, and a change in the optical textures of LCs observed under a POM. A low limit of detection (LOD) of 3.5 nM for AMX was obtained using this sensing system. In addition, this biosensor exhibits high specificity for AMX compared to other antibiotics. Most importantly, the developed LC-based aptasensor was used to successfully analyze AMX in potential application environments, including tap water and river water samples. Therefore, this aptasensor is a simple, sensitive, and label-free platform for the real-time monitoring of AMX in water environments.

## 2. Materials and Methods

### 2.1. Materials and Instruments

The glass slides were provided by Matsunami Glass IND., Ltd. (Osaka, Japan). All the chemicals and reagents were used as received, and deionized (DI) water was used in experiments. In addition, 4-cyano-4′-pentylbiphenyl (5CB) was provided from Tokyo Chemical Industrial Co., Ltd. of Japan. Glutaraldehyde (GA, 50%); (3-aminopropyl) triethoxysilane (APTES, ≥98%), amoxicillin trihydrate (AMX, ≥98%), sulfamethoxazole (≥98%), tetracycline hydrochloride (≥98%), chloramphenicol (≥98%), norfloxacin (≥98%), sulfamethizole (≥99%), ampicillin sodium salt (≥98%), penicillin G sodium salt (≥96%), ethanolamine, dimethyloctadecyl [3-(trimethoxysilyl) propyl] ammonium chloride (DMOAP, 42 wt. %), and phosphate-buffered saline (PBS, pH = 7.4) were procured from Sigma-Aldrich, St. Louis, MO, USA. Amoxicillin aptamer (5′-(NH_2_)-TTA GTT GGG GTT CAG TTG G-3′) was provided by Mbiotech (Hanam, Korea). C_2_H_5_OH, H_2_O_2_ (30%), CH_3_OH, and H_2_SO_4_ (95%) were provided by Daejung Chemicals & Metals Co., Ltd. (Daejung, Korea). Stock antibiotic solutions were prepared in DI water and protected from light.

The surface morphology of the substrates was characterized using an atomic force microscope (AFM; Nanoscope IIIa; Veeco, Santa Barbara, CA, USA) in the tapping mode. A digital camera (DS-2Mv; Tokyo, Japan) mounted on the POM (Eclipse LV100 POL; Nikon, Tokyo, Japan) was used to capture the polarized optical images. The images were taken in transmission mode. The gray-scale intensity (GI) of the images was analyzed using MATLAB software.

### 2.2. Preparation of Modified Glass Slides and Fabrication of LC Cells

The upper and bottom glass slides were prepared following previous studies [27,28]. The LC cells were fabricated as previously described [33,34]. The details are provided in the Supplementary Materials.

### 2.3. Specific Binding of AMX Aptamer and AMX

To prepare for the detection of AMX, 10 μL of AMX aptamer solution at an optimal concentration was dropped onto the GA-modified glass to form a large circular spot, followed by incubating, washing, and drying, as described in the Supplementary Materials. Then, 2 µL of AMX solution at various concentrations was dropped onto the aptamer-modified glass slide and incubated at 25 °C for 2 h in a water-saturated environment. The surface was then washed and dried. The LC cells were then fabricated as described in the Supplementary Materials. To evaluate the selectivity of the biosensor for amoxicillin detection, common interfering antibiotics, such as sulfamethoxazole (SMXZ), tetracycline hydrochloride (TC), chloramphenicol (CL), norfloxacin (NRFX), sulfamethizole (SMZ), ampicillin sodium salt (AMP), and penicillin G sodium salt (PC), were tested under the same optimal conditions at a concentration of 1 µM.

### 2.4. Detection of AMX in Real Water Samples

To evaluate the practicability of the proposed biosensor in a real water environment, AMX was determined in tap water and river water samples. Tap water was obtained from our laboratory, while river water was collected from the Tancheon River, Seongnam, Korea. After filtering, the tap and river water samples were spiked with different concentrations of AMX. The spiked samples were then analyzed using the proposed assay.

## 3. Results

### 3.1. Principle of LC-Based Aptasensor for AMX Detection

**Figure 1:**
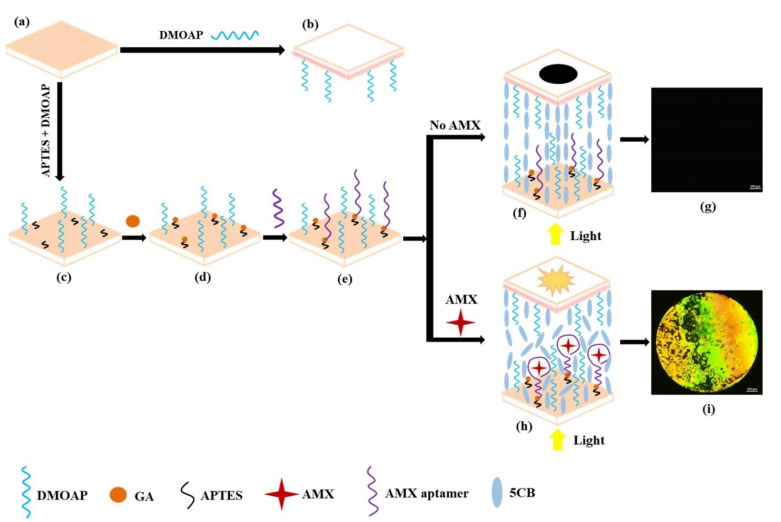
Schematic illustration of the sensing strategy for detecting amoxicillin (AMX) (**a**) Cleaned glass slide; (**b**) upper glass slide; (**c**) the self-assembled monolayer of (3-aminopropyl) triethoxysilane (APTES)/dimethyloctadecyl [3-(trimethoxysilyl) propyl] ammonium chloride (DMOAP) on the glass substrate; (**d**) grafting of glutaraldehyde (GA) onto APTES/DMOAP-modified glass substrate; (**e**) immobilization of the AMX aptamer onto (APTES + GA)/DMOAP-modified glass substrate; (**f**) homeotropic orientation of liquid crystals (LCs) in the absence of AMX; (**g**) polarized optical image of an LC cell in the absence of AMX; (**h**) the conformational change in AMX aptamer after binding to AMX disrupts the homeotropic orientation of LCs; and (**i**) polarized optical image of an LC cell in the presence of AMX. The bright circular spot is caused by the specific binding of aptamer and AMX.

Figure 1 schematically describes the sensing strategy of the proposed LC-based aptasensor for the detection of AMX. The cleaned glass slide (Figure 1a) was decorated with DMOAP and used as an upper glass slide (Figure 1b). A mixed self-assembled film of APTES/DMOAP modified on the glass substrate provides both long alkyl chains for homeotropic alignment of LCs and active amino groups for further coupling of GA (Figure 1c,d). The AMX aptamers (modified with an amino group on the 5′ end) were then immobilized on the (APTES + GA)/DMOAP-modified glass substrate via imine bonds formed from the active aldehyde groups of GA and the amino groups of aptamers (Figure 1e). A proper ratio of DMOAP/APTES/GA and the amount of aptamer immobilized on the glass slide induced a homeotropic orientation in the LCs supported on the modified surface (Figure 1f). With the synergistic effect of the DMOAP molecules modified on the upper glass slide, a dark optical image was observed (Figure 1g). In the presence of AMX, the specific binding of aptamers and AMX molecules disrupted the homeotropic orientation of the LCs (Figure 1h), yielding a bright optical image (Figure 1i). The higher the concentration of AMX, the greater the orientational change in the LC molecules and the brighter the optical POM image. Based on this sensing mechanism, the AMX content in aqueous samples was expected to be determined quantitatively.

### 3.2. Optimization of the Detection Conditions

#### 3.2.1. Effects of the APTES/DMOAP Ratio

APTES and DMOAP were co-immobilized on the glass substrates to induce the homeotropic alignment of LC molecules and provide active amino groups for the further grafting of the AMX aptamer via the bifunctional cross-linking agent GA. The higher the APTES/DMOAP ratio grafted on the glass substrate, the higher the immobilization of aptamers on the glass substrate, and the lower the detection limit of the sensors [18]. However, the surface modification of the glass substrate can significantly affect the orientation behavior of LCs [28,29]. Therefore, the ratio of APTES/DMOAP modified on the glass substrates was first optimized in order to achieve a dark background and achieve the best sensing efficiency. As shown in Figure S1, the POM images of the LC cells gradually changed from bright to dark with a decreasing APTES/DMOAP ratio. Bright POM images were observed when the APTES/DMOAP ratios ≥ 3:1 (*v*/*v*), corresponding to a random orientation of LC molecules (Figure S1a,b). However, completely dark background images were observed when the APTES/DMOAP ratios were reduced to 2:1 or lower, indicating that the LCs exhibited a uniform homeotropic orientation (Figure S1c,d). Therefore, an APTES/DMOAP ratio of 2:1 was employed in subsequent experiments.

#### 3.2.2. Effects of DMOAP Concentrations on the Upper Glass Slides

In addition to the influence of the APTES/DMOAP ratio, the effect of the DMOAP concentration on the upper glass slides on output signals was also investigated. The cleaned glass substrates were coated with various concentrations of DMOAP and used as covering glass slides, while the APTES/DMOAP ratio modified on the bottom glass substrates was fixed at 2:1 (*v*/*v*). As shown in Figure S2a, a bright POM image was observed at a DMOAP concentration of 0%. This demonstrates that without the synergistic effect of the DMOAP molecules modified on the upper glass slide, the LCs exhibited a random orientation due to the lack of alignment agents on the upper glass, resulting in a bright POM image. However, when the DMOAP concentration increased to 0.1% or higher, a uniformly dark optical image was observed (Figure S2b,c). Therefore, a DMOAP concentration of 0.1% was used in all subsequent experiments.

#### 3.2.3. Effects of GA Concentration

The amount of GA grafted onto the APTES/DMOAP-modified glass substrates may also affect the orientation behavior of LCs, thereby affecting the optical appearance of the LCs [29,38]. Therefore, the concentration of GA should also be optimized, while the APTES/DMOAP ratio is maintained at 2:1 (*v*/*v*). As shown in Figure S3a,b, bright images were observed when the GA concentration ≥ 0.1% (*v*/*v*). However, completely dark backgrounds were observed when the GA concentration ≤ 0.01% (*v*/*v*) (Figure S3c,d). Therefore, to ensure a dark background signal and achieve the best sensing efficiency, a GA concentration of 0.01% (*v*/*v*) was used in further experiments.

#### 3.2.4. Effects of AMX Aptamer Concentration

**Figure 2:**
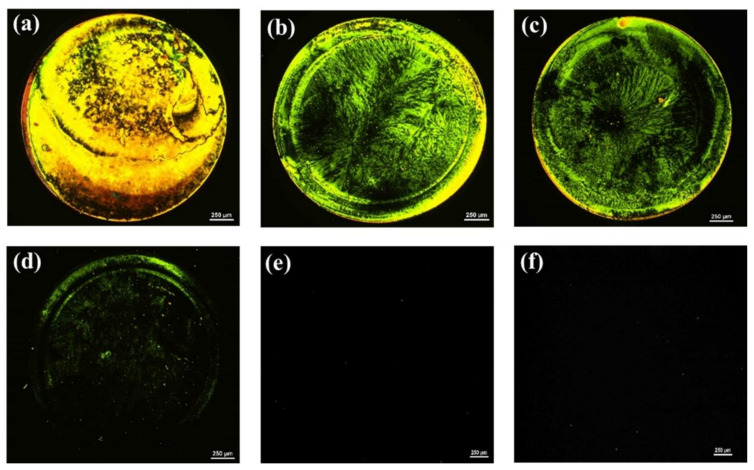
Polarized optical (POM) images of LC cells with 5CB after the modification of the surface of the bottom glass slide with various concentrations of AMX aptamer: (**a**) 1 µM; (**b**) 750 nM; (**c**) 500 nM; (**d**) 250 nM; (**e**) 200 nM; and (**f**) 100 nM. The circular spot shown in the optical images is caused by the presence of AMX aptamers. Scale bar, 250 µm.

Similarly, because the surface topography and homeotropic orientation of LCs are also influenced by the concentration of the aptamer immobilized on the glass substrate, the optimal amount of AMX aptamer to be anchored on the GA-modified glass surface was determined. As shown in Figure 2, the optical appearance of the LC cells changed from bright to dark with a declining aptamer concentration, suggesting that the LCs undergo a transition in their orientation from a random to a homeotropic orientation. While LC cells exhibited bright optical images when the concentration of the aptamer ≥ 250 nM, they appeared uniformly dark when the concentration of aptamer ≤ 200 nM. This indicated that the orientation of LCs was strongly affected by the amount of AMX aptamer; the higher the amount of aptamer immobilized on the surface, the greater the disturbance of the LC alignment, and the brighter the obtained optical images. Therefore, to ensure the homeotropic orientation of LCs and provide as much aptamer on the glass substrate as needed, an AMX aptamer concentration of 200 nM was used for the subsequent detection of AMX.

### 3.3. Feasibility of the LC-Based Aptasensor for AMX Detection

**Figure 3:**
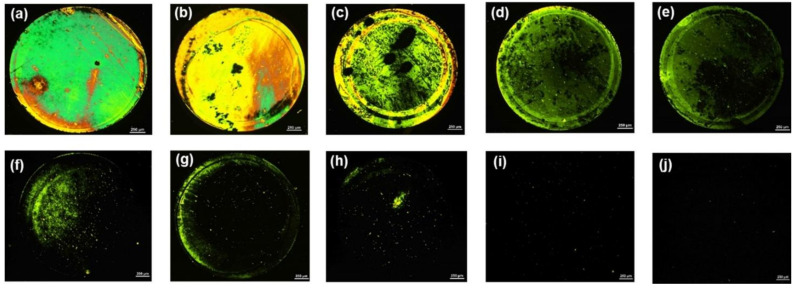
POM images of LC cells with AMX at the concentration of: (**a**) 1 µM; (**b**) 800 nM; (**c**) 600 nM; (**d**) 400 nM; (**e**) 200 nM; (**f**) 100 nM; (**g**) 50 nM; (**h**) 10 nM; (**i**) 5 nM; and (**j**) 0 nM. Scale bar, 250 µm.

Next, we examined the feasibility of the aptasensor developed in this study for the detection of AMX under optimal experimental conditions. An LC cell with its bottom glass substrate incubated with 1 µM AMX solution was fabricated and examined. LCs showed a bright circular spot in the region where the AMX solution was dropped, but it remained dark outside the spotted circle (Figure 3a). This indicated that the aptamers immobilized on the surface did not affect the homeotropic orientation of LCs, with the bright optical signal caused only by the specific binding of AMX to the aptamer. This phenomenon verified that the binding of the AMX aptamer with AMX disrupted the homeotropic alignment of LCs, resulting in a transition from a dark to a bright optical image of the LC cells. This result demonstrates that AMX can be monitored using the proposed method.

**Figure 4:**
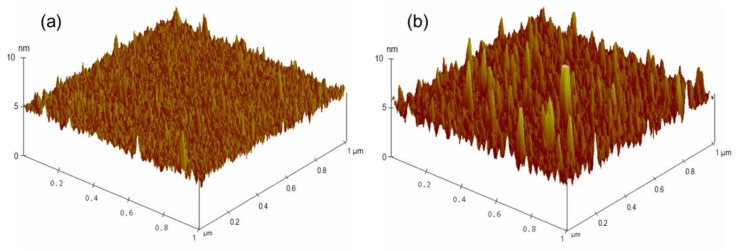
Three-dimensional AFM images of substrates immobilized with 200 nM AMX aptamer (**a**), and upon incubation of 800 nM amoxicillin (**b**).

After confirming the feasibility of the developed sensing system, the limit of detection of this biosensor for AMX was investigated by decreasing the concentration of AMX. A series of LC cells with their bottom glass substrates incubated with various AMX concentrations ranging from 0 nM to 800 nM was constructed and examined. For AMX concentrations ranging from 50 nM to 800 nM, bright circular spots on the POM images of the LC cells were observed (Figure 3b–g), indicating that the AMX molecules were captured by the AMX aptamer and induced the orientational transition of the LCs supported on the surface from a homeotropic to a random orientation. The brightness of the circular spot gradually decreased with a decline in the AMX concentration. When the concentration of AMX was reduced to 10 nM, some bright spots appeared in the area where the AMX solution was dropped (Figure 3h), indicating that a small amount of AMX could somewhat induce the orientational transition of LCs. With a further decrease in the AMX concentration to 5 nM, an almost uniformly dark image was observed (Figure 3i), suggesting that the amount of captured AMX was not sufficient to induce the orientational transition of LCs. Changes in the surface morphology of the substrate were attributed to the specific interactions between AMX molecules and AMX aptamers, further inducing the orientational transition of LCs, resulting in a change in optical signal. This conclusion was confirmed by the AFM images. A comparison of the surface morphology of the aptamer-immobilized glass substrates before and after incubation with AMX is shown in Figure 4. The surface morphology of the aptamer-immobilized glass substrate (Figure 4a) changed markedly after binding with AMX (Figure 4b). These results suggested that a minimum AMX concentration of 10 nM was required to trigger changes in the orientation of LCs from homeotropic to random.

**Figure 5:**
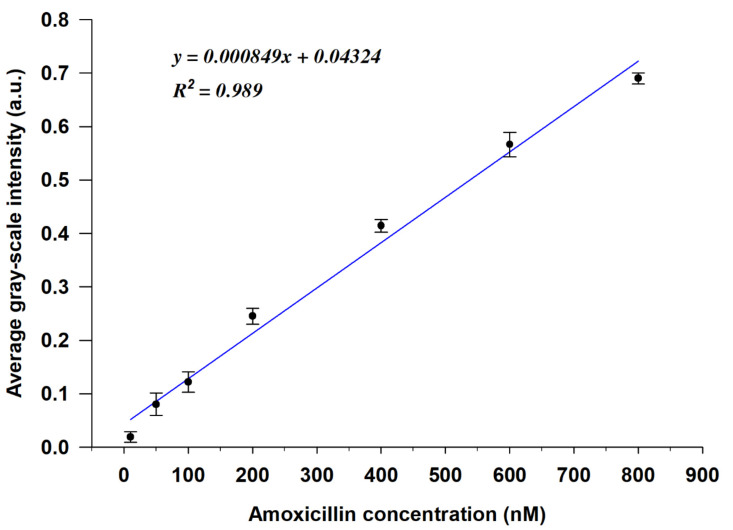
Calibration curve of the average gray-scale intensities (GIs) of the POM images vs. the concentration of AMX (y is the average GI of the POM image, x is the concentration of AMX, R^2^ is the linear correlation coefficient) (*n* = 3).

To further investigate the quantitative performance of the LC-based aptasensor, we evaluated the correlation between the AMX concentration and the GI of the POM images calculated using MATLAB software (the MATLAB code is given in the Supplementary Materials). As shown in Figure 5, the GIs of the optical images gradually increased with increasing AMX concentration, and the linear regression curve exhibited a linear relationship between the average GI and the AMX concentration in the range of 10 nM to 800 nM, with a high linear correlation coefficient (R^2^) of 0.989. The detection limit of AMX was found to be 3.5 nM, which was calculated using the formula 3α/slope [39,40]. However, when the AMX concentration ≥ 800 nM, the GIs of the optical images tended to saturate (Figure S4). Compared with the existing assays for amoxicillin detection, the method that we developed in the present study exhibits relatively high sensitivity with a competitive LOD, as shown in Table 1.

### 3.4. Sensor Specificity

**Figure 6:**
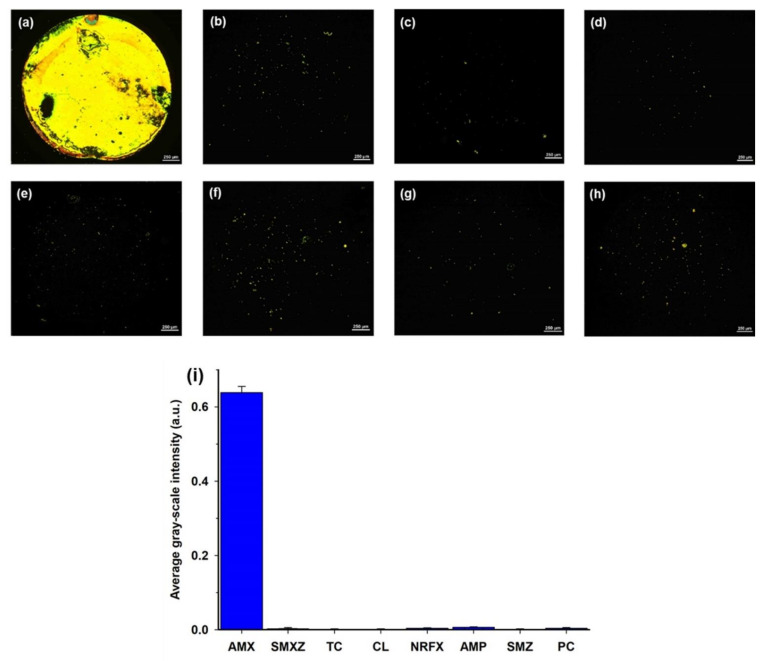
POM images of LC cells in which the bottom glass substrates were incubated with different antibiotic solutions: (**a**) 0.8 µM AMX; (**b**) 1 µM SMXZ; (**c**) 1 µM TC; (**d**) 1 µM CL; (**e**) 1 µM NRFX; (**f**) 1 µM AMP; (**g**) 1 µM SMZ; and (**h**) 1 µM PC. (**i**) Average GI of optical images caused by amoxicillin (0.8 µM) and other control antibiotics (1 µM). Scale bar, 250 µm.

In addition to the sensitivity, the specificity of the LC assay was also evaluated. Several commonly used antibiotics, such as SMXZ, TC, CL, NRFX, SMZ, AMP, and PC, that have similar structures with AMX and are difficult to distinguish, were selected as interferences [1,9,15]. A series of LC cells with their bottom glass substrates incubated with different antibiotic solutions was constructed and examined. As shown in Figure 6a, the optical image appeared bright when the AMX solution (0.8 μM) was incubated on the aptamer-modified glass slides. In contrast, the optical images appeared almost completely dark when other antibiotic solutions (1 μM) were incubated on the aptamer-modified glass slides; only some weak bright spots were observed (Figure 6b–h). Moreover, the average GI of the optical image for AMX was significantly higher than that of other antibiotics (Figure 6i). These results indicate the good specificity of the proposed LC-based aptasensor towards AMX.

### 3.5. Real Sample Analysis

The applicability of the proposed aptasensor for the determination of AMX in real water samples, including tap water and river water, was tested. The results are shown in Figures S5 and S6 in the Supplementary Materials. Based on the calibration curve in Figure 5, the found concentrations of AMX in spiked tap water and river water were calculated. As shown in Table 2, the recovery test indicated recoveries between 91.16 and 107.58%, with a relative standard deviation of less than 8%, indicating the high accuracy of the developed aptasensor for AMX detection. These results demonstrate that this LC-based aptamer sensing platform is reliable and feasible for the monitoring of AMX in environmental water samples.

## 4. Conclusions

In this study, an LC-based biosensing strategy using a specific DNA aptamer as a target recognition probe was developed to detect amoxicillin in aqueous media. The detection principle is based on the specific binding of AMX molecules to the DNA aptamer immobilized on the LC cell, which disrupts the homeotropic alignment of LCs, resulting in a bright optical image observed under a POM. Using this sensing strategy, under optimal conditions, AMX could be quantified in the range of 10 nM to 800 nM with a minimum detection limit of 3.5 nM. In addition, this biosensor exhibited high specificity for AMX compared to other antibiotics. Moreover, the developed biosensor was successfully applied to tap water and river water. This proposed sensing strategy offers a simple, convenient, label-free, and sensitive method for the detection of AMX in aqueous solutions.

## Figures and Tables

**Table 1:** Comparison with various methods for the detection of amoxicillin.

Methods (Materials)	Linear Ranges	Detection Limit	Reference
Electrochemistry (TiO_2_-g-C_3_N_4_@Au NPs) ^a^	0.5–3 nM	0.2 nM	[1]
Electrochemistry (AuNP-PdNP-ErGO) ^b^	30–350 μM	9 μM	[7]
Photoluminescence (MIP-CdTeQDs) ^c^	0.547–163.8 μM	0.383 nM	[8]
Fluorescence (B-CQDs) ^d^	1.43–429.12 μM	825 nM	[10]
Electrochemistry (P6LC/CdTe QDs) ^e^	0.9–69 µM	50 nM	[12]
HPLC ^f^	0.137–1368 µM	43.8 nM	[13]
Chemosensor (hybrid AMO-MIP) ^g^	100 pM–8 µM	73 pM	[15]
Colorimetry (Qt AgNPs) ^h^	10–95 µM	4.46 µM	[16]
Liquid crystal-based aptasensor	10–800 nM	3.5 nM	Present study

^a^ TiO_2_-g-C_3_N_4_@Au NPs, titanium dioxide/graphite phase carbon nitrite/gold nanoparticles (NPs). ^b^ AuNP-PdNP-ErGO, gold nanoparticles/palladium nanoparticles decorated electrochemically with reduced graphene oxide. ^c^ MIP-CdTeQDs, molecularly imprinted polymer (MIP)-coated cadmium telluride (CdTe) quantum dots. ^d^ B-CQDs, boron-doped carbon quantum dots. ^e^ P6LC/CdTe QDs, Printex 6L Carbon/CdTe quantum dots. ^f^ HPLC, high-performance liquid chromatography. ^g^ Hybrid AMO-MIP, hybrid organic–inorganic MIP selective towards amoxicillin. ^h^ Qt AgNPs, quercetagetin-stabilized silver NPs.

**Table 2:** Determination of AMX in spiked real water samples (*n* = 3).

Samples	Spiked AMX (nM)	Found AMX (nM)	Recovery (%)	Relative Standard Deviation (RSD) (%)
Tap water 1	50	46.175	92.35	4.71
Tap water 2	400	430.35	107.58	2.37
Tap water 3	800	758.45	94.8	2.18
River water 1	50	45.58	91.16	4.96
River water 2	400	386.76	96.69	7.91
River water 3	800	754.11	94.26	1.57

## Data Availability

Not applicable.

## Supplementary Materials

The following are available online at https://www.mdpi.com/article/10.3390/mi12040370/s1. Figure S1. POM images of LC cells with 5CB in which the surface of the bottom glass slide is modified with different ratios (*v*/*v*) of APTES/DMOAP: (**a**) 5:1; (**b**) 3:1; (**c**) 2:1 and (**d**) 1:1. Scale bar, 200 µm. Figure S2. POM images of LC cells with 5CB in which the bottom glass slide is modified with APTES/DMOAP (2:1) while the covering glass slide is modified with DMOAP at different concentrations (*v*/*v*): (**a**) 0%; (**b**) 0.1%; and (**c**) 0.2%. Scale bar, 200 µm. Figure S3. POM images of LC cells with 5CB in which the surface of the bottom glass slide is grafted with various concentrations of GA (APTES/DMOAP = 2:1): (**a**) 1%; (**b**) 0.1%; (**c**) 0.01% and (**d**) 0.001%. The covering glass slide is modified with 0.1% DMOAP. Scale bar, 200 µm. Figure S4. Relationship between the average gray-scale intensity of the optical images and the concentrations of AMX. When the concentration of AMX was over 800 nM, the optical signal of the LC cells was saturated. Figure S5. POM images of LC cells with 5CB in the presence of different concentrations of AMX in spiked tap water samples: (**a**) 50 nM, (**b**) 400 nM, and (**c**) 800 nM. Scale bar, 250 µm. Figure S6. POM images of LC cells with 5CB in the presence of different concentrations of AMX in spiked river water samples: (**a**) 50 nM, (**b**) 400 nM, and (**c**) 800 nM. Scale bar, 250 µm.
