# Supplementary material for: A Label-Free Liquid Crystal Biosensor Based on Specific DNA Aptamer Probes for Sensitive Detection of Amoxicillin Antibiotic
Source: Micromachines (Basel). 2021 Mar 30;12(4):370. doi: 10.3390/mi12040370 (PMC8065461; doi:10.3390/mi12040370)
Supplement: Supplementary file 1 [file micromachines-12-00370-s001.pdf]

Supplementary Material

A Label-Free Liquid Crystal Biosensor Based on Specific DNA Aptamer Probes for Sensitive Detection of Amoxicillin Antibiotic

Duy Khiem Nguyen and Chang-Hyun Jang

1. Preparation of modified glass slides and fabrication of LC cells

1.1. Cleaning of glass slides

Glass slides were cut into 2.5 cm × 1 cm pieces and immersed in a freshly prepared piranha solution (70% H_2SO_4 and 30% H_2O_2, v/v) at 80 °C for 2 h in a nitrogen-rich atmosphere to remove all organic contaminants on the surface of glass slides. After cooling, the slides were rinsed turn-wise in DI water, ethanol, and methanol to remove the residual acid, followed by drying under nitrogen flow and heating in an oven at 120 °C overnight. Hydroxyl groups were formed on the surface of the glass slides after piranha treatment.

1.2. Preparation of covering glass slides

The cleaned glass slides were immersed in an aqueous solution containing 0.1% (v/v) DMOAP at room temperature for 1 h, rinsed several times with DI water, dried with nitrogen flow, and heated at 120 °C for 1 h. The DMOAP-modified glass slides were then utilized as upper glass slides in the fabrication of LC cells in subsequent experiments.

1.3. Preparation of bottom glass slides

1.3.1. Preparation of GA-modified glass slides

First, the cleaned glass slides were immersed in an ethanol solution containing a mixture of 0.8% (v/v) APTES and 0.4% (v/v) DMOAP at 80 °C for 1 h. After washing several times with ethanol and DI water, the glass slides were dried under nitrogen flow and heated at 120 °C for 1 h. Subsequently, the APTES/DMOAP-modified glass slides were immersed in an aqueous solution containing 0.001% (v/v) GA at room temperature for 30 min, followed by washing several times with DI water and drying under nitrogen flow. The GA-modified glass slides were stored at 4°C until further use.

1.3.2. Immobilization of AMX aptamers onto GA-modified glass slides

AMX solutions of various concentrations were prepared by diluting a 100 μM AMX stock solution with 0.01 mol L^{-1} PBS buffer (pH 7.4, 138 mM NaCl, 2.7 mM KCl). A volume of 2 μL of AMX aptamer solution at each concentration was dropped onto the surface of GA-modified glass slides to form circular spots, and then incubated in an oven at 25 °C for 2 h in a water-saturated environment. After incubation, the glass slides were rinsed successively with PBS buffer (pH 7.4) and DI water several times to remove the unbound AMX aptamer, and then dried under a nitrogen stream. This resulted in the immobilization of the AMX aptamer on the glass slides with a circular spot of

approximately 2.2 mm. To block the unreacted aldehyde groups (from GA), the AMX aptamer-immobilized glass slides were immersed in 20 mM ethanolamine aqueous solution at room temperature (~25 °C) for 30 min, followed by washing with excess DI water and drying under nitrogen flow. The AMX aptamer-immobilized glass slides served as bottom glass slides in the fabrication of LC biosensors.

1.4. Fabrication of LC cells

To fabricate the LC cells, the upper glass slides modified with DMOAP and the bottom glass slides modified with different reagents were paired face to face, spaced with a thin polyester film (thickness ~10 μm), and held together with two small binder clips. The 5CB was heated on a hot plate to a temperature of around 40 °C, corresponding to its isotropic phase, before injecting into the gap between the two glass slides using a micropipette. Finally, the LC cells were slowly cooled to room temperature (~25 °C) in the nematic phase of 5CB prior to characterization.

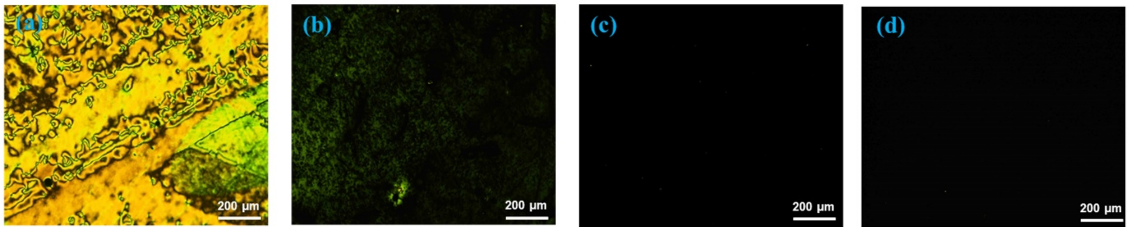

Figure S1. POM images of LC cells with 5CB in which the surface of the bottom glass slide is modified with different ratios (v/v) of APTES/DMOAP: (**a**) 5:1; (**b**) 3:1; (**c**) 2:1 and (**d**) 1:1. Scale bar, 200 μm.

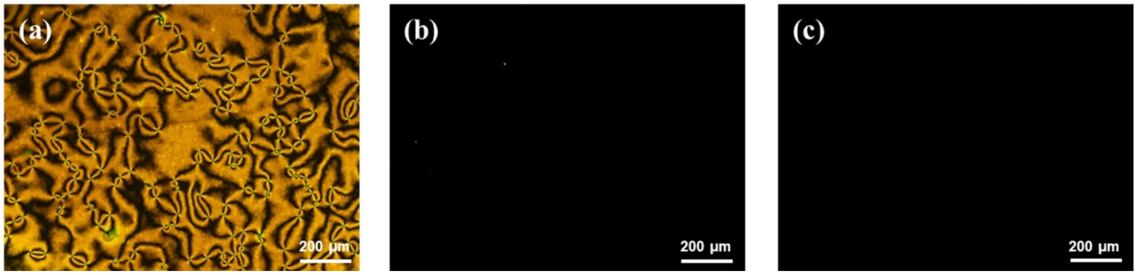

Figure S2. POM images of LC cells with 5CB in which the bottom glass slide is modified with APTES/DMOAP (2:1) while the covering glass slide is modified with DMOAP at different concentrations (v/v): (**a**) 0 %; (**b**) 0.1%; and (**c**) 0.2%. Scale bar, 200 μm.

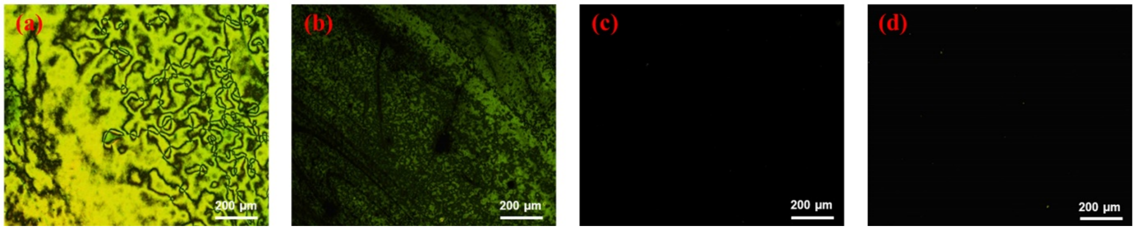

Figure S3. POM images of LC cells with 5CB in which the surface of the bottom glass slide is grafted with various concentrations of GA (APTES/DMOAP = 2:1): (**a**) 1%; (**b**) 0.1%; (**c**) 0.01% and (**d**) 0.001%. The covering glass slide is modified with 0.1% DMOAP. Scale bar, 200 μm.

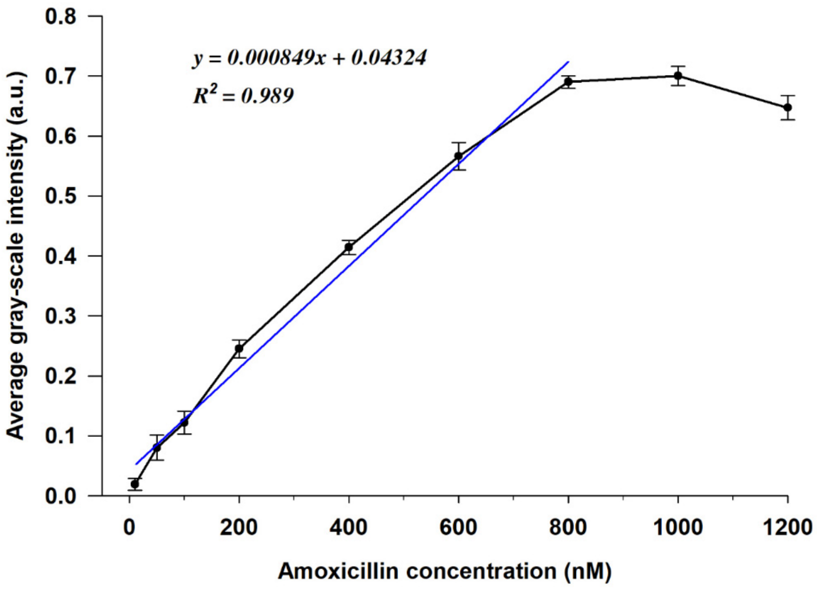

Figure S4. Relationship between the average gray-scale intensity of the optical images and the concentrations of AMX. When the concentration of AMX was over 800 nM, the optical signal of the LC cells was saturated.

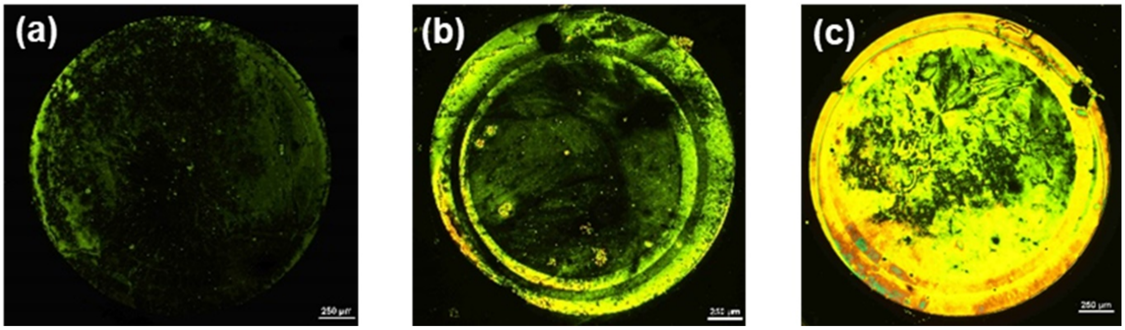

Figure S5. POM images of LC cells with 5CB in the presence of different concentrations of AMX in spiked tap water samples: (**a**) 50 nM, (**b**) 400 nM, and (**c**) 800 nM. Scale bar, 250 μm.

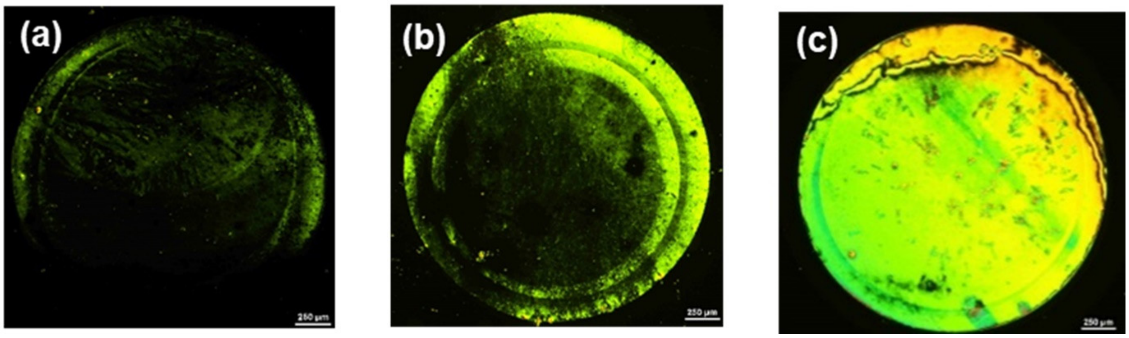

Figure S6. POM images of LC cells with 5CB in the presence of different concentrations of AMX in spiked river water samples: (**a**) 50 nM, (**b**) 400 nM, and (**c**) 800 nM. Scale bar, 250 μm.

MATLAB code used for calculation of gray-scale intensity of the POM images

```
clear all

close all

clc

% Directory of image folder

file_path = 'images/';

img_path_list = dir(strcat(file_path,'*.jpg'));

% Num of images

img_num = length(img_path_list);

% Zero array

Result_Value = zeros(img_num,1);

addpath(file_path);

i = 1;

fprintf('Num of images: %d \n', img_num);

while i <= img_num

    % Read and convert image from color to grayscale

    Orig_Picture = imread (img_path_list(i).name);

    % Threshold for zero pixel

    Gray_Picture = im2bw(Orig_Picture,0.15);

    % Count num of none zero pixels

    Light_Pixel_Num = nnz (Gray_Picture);

    % Size of gray image

    [m, n] = size(Gray_Picture);

    % Total pixels

    Picture_Pixel_Num = m * n;

    % Intensity value

    Result_Value(i,1) = Light_Pixel_Num / Picture_Pixel_Num;

    % Print output value

    fprintf('The intensity of image %s is %f \n', img_path_list(i).name, Result_Value(i,1));
```

```
    i= i + 1;
end
```
